# DNA-demethylation by DAC induces *MAGE* expression and MAGE-specific T cell reactivity against tumors but also healthy cell subsets

**DOI:** 10.1016/j.omton.2025.201018

**Published:** 2025-07-17

**Authors:** Marije A.J. de Rooij, Miranda H. Meeuwsen, Anne K. Wouters, Dennis F.G. Remst, Renate S. Hagedoorn, Dirk M. van der Steen, Els M.E. Verdegaal, Tassilo L.A. Wachsmann, J.H. Frederik Falkenburg, Mirjam H.M. Heemskerk

**Affiliations:** 1 Department of Hematology, Leiden University Medical Center, Leiden 2333ZA, the Netherlands; 2 Department of Medical Oncology, Oncode Institute, Leiden University Medical Center, Leiden 2333ZA, the Netherlands

**Keywords:** MT: Regular Issue, CD8^+^ T cell, TCR, DAC, MAGE, DNA-demethylation, solid tumors

## Abstract

Cancer testis antigens (CTAs) can be expressed in tumors, whereas expression is silenced in normal tissue except for the immune-privileged testis. This quasi-tumor-restricted expression makes CTAs attractive targets for T cell receptor (TCR) gene therapy. However, CTA-specific TCR gene therapy is only applicable for tumors with substantial and homogeneous CTA expression. To increase the number of patients eligible for CTA-specific TCR gene therapy, CTA expression can be upregulated with DNA-demethylating agents like 5-aza-2′-deoxycytidine (DAC). Here, we studied the effect of DAC on the recognition of a wide range of tumor cells by TCR-engineered T cells specific for the CTAs MAGE-A1, MAGE-A3/A6, or MAGE-A9. DAC treatment strongly increased *MAGE* expression in most tumor cell lines tested and strongly induced or improved recognition by MAGE-specific TCR-engineered T cells. However, *MAGE* upregulation was not limited to tumor cells but also occurred in healthy cells, resulting in MAGE-specific T cell reactivity against proliferating T and B cells. Overall, these results underscore the potential of DAC treatment to induce *MAGE* expression in tumor cells and to increase their sensitivity for MAGE-specific T cell therapy. However, DAC treatment can potentially result in on-target off-tumor reactivity, warranting careful consideration when using DAC as sensitizing strategy prior to adoptive transfer of CTA-specific T cells.

## Graphical abstract

**Figure:**
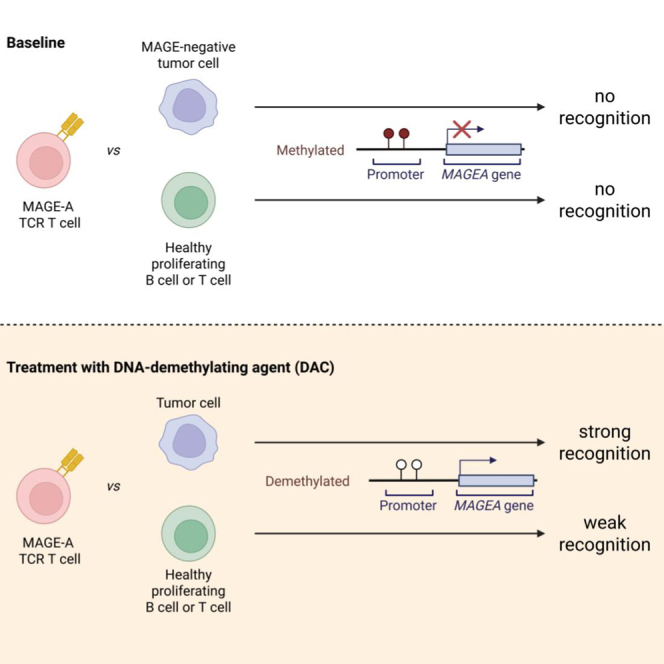

## Teaser

Treatment with DNA-demethylating agents induces MAGE antigen expression in tumor cells, sensitizing them to recognition by MAGE-specific T cells. In this publication by de Rooij and colleagues, DNA-demethylating agents also induced MAGE expression in proliferating B cells and T cells, suggesting potential safety risks of this approach.

## Introduction

Cancer testis antigens (CTAs) have been described as ideal targets for T cell receptor (TCR) gene therapy since they are highly expressed in multiple different tumor types and silenced in healthy tissue except for the immune-privileged testis. However, the application of CTA-specific TCR gene therapy is typically restricted to those patients who display high tumoral expression of CTA genes, and heterogeneous expression within tumors can limit efficacy. Treatment with the DNA-demethylating agent 5-aza-2′-deoxycytidine (DAC) can artificially induce the expression of CTA genes in a variety of different tumor types^1^^,^^2^^,^^3^^,^^4^^,^^5^^,^^6^^,^^7^ and can reduce intratumor heterogeneity to enhance T-cell-mediated tumor killing and prevent tumor escape.^8^^,^^9^^,^^10^ In addition, expression of other molecules important for T cell-tumor cell interaction can be induced by DAC treatment, such as mediators of interferon (IFN) signaling, major histocompatibility complexes (MHCs), adhesion molecules (such as ICAM-I), immune proteasome subunits (PSMB8 and PSMB9), and ER transporters (TAP1).^11^^,^^12^^,^^13^ These findings make treatment with DAC a promising strategy to sensitize tumor cells for T-cell-mediated cytotoxicity in TCR gene therapy.

DAC, also known as decitabine, is a European Medicines Agency (EMA) and Food and Drug Administration (FDA)-approved treatment for myelodysplastic syndrome and acute myeloid leukemia.^14^^,^^15^^,^^16^ In this setting, DAC is administered at high concentrations to interfere with normal DNA synthesis, causing reduced cell-cycle activity or apoptosis.^17^ At lower concentrations, DAC does not interfere with DNA synthesis, whereas it exerts a DNA-demethylating function.^18^^,^^19^ DAC-induced DNA-demethylation depends on incorporation of DAC into the DNA during DNA replication and therefore preferentially occurs in proliferating cells.^20^ Several studies reported that the demethylating effect of DAC is limited to malignant cells due to their high proliferative turnover and that DAC does not affect normal tissues including several epithelial cell lines, melanocytes, fibroblasts, keratinocytes, and peripheral blood lymphocytes.^2^^,^^3^^,^^21^ Nevertheless, incorporation of DAC can principally occur during the cell cycle of every cell type. It remains questionable whether DAC treatment prior to TCR gene therapy might induce on-target off-tumor toxicity of CTA-specific TCR gene therapy against highly proliferating normal cell subsets.

This study aimed to investigate the effectiveness and safety of DAC treatment in combination with CTA-specific TCR-engineered T cell (TCR T cell) therapy *in vitro*. Effector functions of T cells transduced to express previously identified TCRs targeting MAGE-A1 in the context of HLA-A2 (MAGE-A1 HLA-A2), MAGE-A3/-A6 in the context of HLA-B35 (MAGE-A3/-A6 HLA-B35), and MAGE-A9 in the context of HLA-A1 (MAGE-A9 HLA-A1) were analyzed after co-culture with multiple different DAC-treated malignant cell lines and healthy cell subsets.^22^ Strong anti-tumor reactivity of the CTA-specific TCR T cells against different tumor cell lines correlated with markedly induced expression of *MAGEA1*, *MAGEA3/A6*, and *MAGEA9* by DAC treatment. However, DAC treatment also induced on-target off-tumor toxicity against *in vitro* activated T and B cells, which correlated with increased CTA gene expression. Overall, these results confirm that tumor cells strongly upregulate *MAGE* after DAC treatment, leading to enhanced MAGE-specific T cell-mediated reactivity. Nevertheless, toxicity risks of potent MAGE-specific TCR T cells against highly proliferating cells, such as activated T and B cells, should be taken into consideration when combining MAGE-specific TCR T cell therapy with administration of DAC.

## Results

### DAC strongly induces *MAGE* expression in a variety of different tumor types

**Figure 1:**
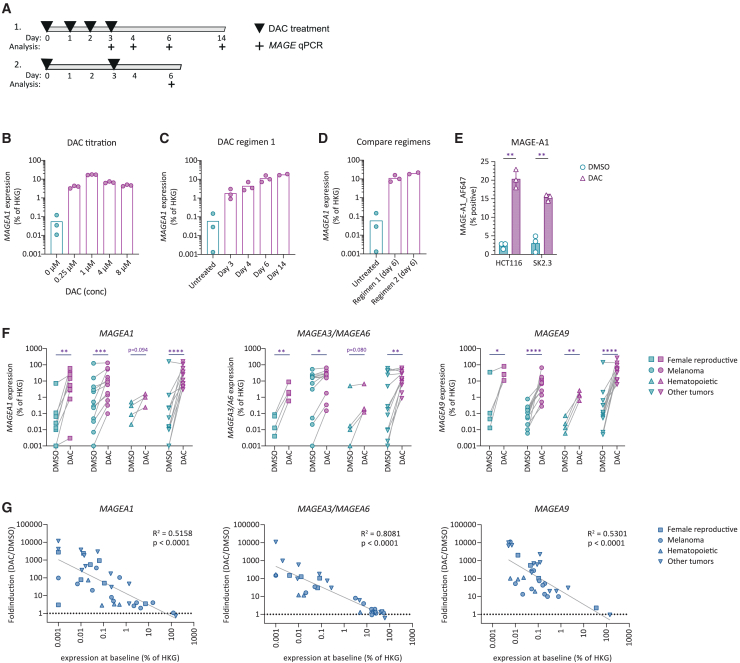
DAC treatment enhances *MAGE* expression in tumor cell lines (A) Different treatment schemes of melanoma cell line SK2.3 were tested followed by analysis (+) of *MAGE* expression. (B) *MAGEA1* expression in SK2.3 was measured by qPCR after exposure to DAC at indicated different concentrations. (C) *MAGEA1* expression in SK2.3 over time after induction with DAC in a concentration of 1 μM following treatment regimen 1. (D) Comparison of *MAGEA1* induction on day 6 after start of treatment with 1.0 μM DAC following regimen 1 or 2. In (B–D), symbols represent technical replicates. (C and D) Data derived from the same experiment. (E) MAGE-A1 protein expression as detected by flow cytometry in indicated cell lines following DAC treatment. Statistics depict paired t tests on data generated from three independent experiments. (F) Expression of *MAGEA1*, *MAGEA3*/*MAGEA6*, or *MAGEA9* after treatment with 1.0 μM DAC following regimen 2 in cell lines of different tissue origins as indicated by symbols. Data points depict averaged triplicate values of *MAGE* gene expression of individual cell lines as determined by qPCR. Refer to Table 1 for individual data points. Statistics depict multiple t tests on log-transformed data of DMSO- to DAC-treated cell lines derived from indicated tissue origins. (G) Linear regression analysis on x and y log-transformed data plotting expression at baseline versus fold induction after DAC treatment for indicated *MAGE* genes.

To induce expression of *MAGE* genes *in vitro* by DAC treatment, we first tested different treatment regimens on the melanoma cell line SK2.3 that naturally expresses very low levels of *MAGEA1*. First, SK2.3 was treated on four consecutive days with different concentrations of DAC and analyzed for changes in *MAGEA1* mRNA expression by qPCR (qPCR) (Figure 1A). DAC treatment induced strong upregulation of *MAGEA1* expression from a concentration of 0.25 μM, while a concentration of 1 μM resulted in the strongest upregulation (Figure 1B). Further increase to 4 μM or 8 μM did not result in any further increase in expression of *MAGEA1*. After stopping DAC treatment on day 3, *MAGEA1* expression increased even further (Figure 1C). The increase in *MAGEA1* expression persisted until day 14 after start of the treatment, indicating a durable effect of DAC treatment on *MAGEA1* gene expression (Figure 1C). DAC treatment every 3 days (regimen 2) was equally efficient in inducing *MAGEA1* expression as was daily treatment for four consecutive days (Figure 1D). For all consecutive experiments we therefore treated healthy or tumor cells twice on day 0 and day 3 with 0.5 μM or 1.0 μM and used DAC-treated cells for follow-up experiments on day 6 after induction. To assess whether DAC-induced upregulation of mRNA expression correlates with an increase of protein expression, we performed intracellular detection of MAGE protein using flow cytometry. DAC treatment induced protein expression in both cell lines tested, HCT116 and SK2.3, corresponding with an increase in *MAGEA1* gene expression (Figures 1E and S1).

We continued to assess the effect of DAC treatment on the expression of *MAGEA1*, *MAGEA3/MAGEA6*, and *MAGEA9* in a broad panel consisting of 37 cell lines originating from female reproductive organ tumors, melanoma, hematopoietic, or other cancers (Figure 1F; Table 1). DAC treatment induced strong upregulation of *MAGEA1*, *MAGEA3/MAGEA6*, and *MAGEA9* in almost all cell lines tested. The degree of induction showed an inverse correlation with baseline expression levels (Figure 1G). DAC induced up to 10,000-fold induction of *MAGE* genes in tumor cells that displayed low or absent *MAGE* expression at baseline, whereas in tumors with high baseline expression *MAGE* expression was less affected by DAC treatment. These results demonstrate that DAC treatment can induce substantial expression of *MAGE* family of genes in both *MAGE*-negative and *MAGE*-low tumor cells.

**Table 1:** *MAGEA1*, *MAGEA3/A6*, and *MAGEA9* expression as measure by qPCR indicated as % expression relative to housekeeping gene (HKG) in cell lines of various origins after DMSO or DAC treatment

			*MAGEA1* (% of HKG)		*MAGEA3/A6* (% of HKG)		*MAGEA9* (% of HKG)	
Category	Tumor type	Cell line	DMSO	DAC	DMSO	DAC	DMSO	DAC
Female reproductive	cervix carcinoma	Ca Ski	0.001	2.768	0.013	1.640	0.013	26.152
	mammary carcinoma	CAMA1	0.010	0.781	0.004	0.592	0.105	10.882
	mammary carcinoma	BT-549	0.026	14.359	0.085	8.839	0.046	25.000
	ovarian carcinoma	A2780 AD	0.065	60.570	N.I.	N.I.	N.I.	N.I.
	ovarian carcinoma	JC	0.109	5.504	N.I.	N.I.	N.I.	N.I.
	ovarian carcinoma	COV 362.4	0.000	2.991	N.I.	N.I.	N.I.	N.I.
	ovarian carcinoma	COV318	0.001	0.003	N.I.	N.I.	N.I.	N.I.
	ovarian carcinoma	OVCAR3	0.013	30.921	N.I.	N.I.	N.I.	N.I.
	ovarian carcinoma	COV434	7.179	25.000	0.069	2.062	35.355	81.225
Melanoma	melanoma	MI 3046/2	0.515	53.589	3.125	25.000	0.006	66.204
	melanoma	SK2.3	0.048	17.274	22.445	43.966	0.052	14.031
	melanoma	MEL06.04	0.001	0.098	0.001	0.148	0.010	0.515
	melanoma	MEL09.10	0.276	1.360	25.000	28.717	0.052	1.360
	melanoma	MEL19.05	0.007	0.317	0.021	0.340	0.021	0.276
	melanoma	MEL08.11	0.032	0.635	0.052	1.795	0.195	10.153
	melanoma	MEL13.07	2.210	5.831	16.117	19.615	0.191	2.538
	melanoma	MEL15.02	2.916	11.663	15.932	30.779	0.064	17.678
	melanoma	MEL13.11	15.389	61.557	7.179	28.717	0.112	8.247
	melanoma	MEL15.09b	123.114	131.951	18.946	26.794	0.159	6.699
	melanoma	MEL08.04	4.419	8.839	16.494	15.389	0.781	7.695
	melanoma	MEL06.24	0.224	1.795	53.589	65.975	0.258	2.538
Hematopoietic	AML	AML02	0.298	0.989	0.010	0.120	0.023	2.598
	AML	AML03	0.021	1.568	0.000	0.168	0.073	1,408
	multiple myeloma	UM6	0.085	0.242	0.017	0.199	0.006	0.611
	multiple myeloma	UM9	0.508	1.606	5.059	6.675	0.013	1.192
Other	bile duct carcinoma	EGI-1	0.224	6.699	0.010	5.831	0.007	50.000
	prostate carcinoma	C4-2B4	159.107	114.472	23.899	29.267	0.321	6.228
	prostate carcinoma	DU145	0.014	25.000	0.001	10.882	0.064	43.528
	lung Carcinoma	H292	0.001	11.663	0.182	13.397	0.005	50.000
	Ewing sarcoma	TC71	1.360	61.557	0.276	8.247	2.062	61.557
	osteosarcoma	SJSA-1	0.001	4.123	0.002	1.924	0.005	32.988
	rhabdomyosarcoma	TE671	0.016	7.179	40.613	53.589	141.421	131.951
	renal cell carcinoma	RCC1851	0.011	2.956	0.009	0.867	0.052	10.996
	neuroblastoma	SJNB-8	0.209	35.355	4.737	28.717	0.091	53.589
	neuroblastoma	SK-N-BE	1,360	4.737	61.557	37.893	0.635	12.500
	colon carcinoma	HCT116	0.055	161.888	45.850	65.293	0.120	266.660
	colon carcinoma	WiDr	0.010	37.242	0.082	14.559	0.122	95.264

See also Figure 1F.

AML, acute myeloid leukemia; N.I., not included in analysis.

### DAC treatment sensitizes tumor cell lines to recognition by MAGE-specific TCR-transduced T cells

After having demonstrated that DAC treatment induced strong upregulation of *MAGE* genes in a wide array of tumor cells, we continued to test whether increase in *MAGE* expression would sensitize tumor cells toward recognition by MAGE-specific TCR-transduced T cells. We generated T cells expressing previously characterized TCRs specific for peptides derived from MAGE-A1 presented in HLA-A∗02:01, MAGE-A3/A6 in HLA-B∗35:01, or MAGE-A9 in HLA-A∗01:01, respectively.^22^ Primary human CD8^+^ T cells were isolated from healthy donor peripheral blood mononuclear cells, activated, transduced, and enriched for transgenic TCR expression (Figure S2A). All generated TCR T cell products demonstrated antigen-specific reactivity (Figure S2B).

**Figure 2:**
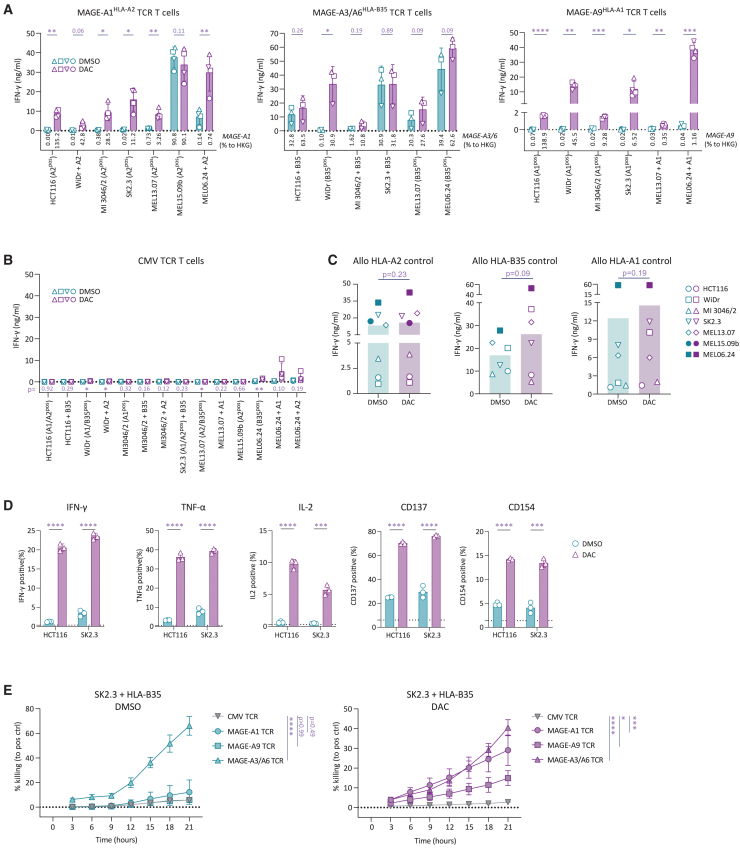
DAC treatment improves anti-tumor reactivity of MAGE TCR T cells (A) Reactivity of purified indicated MAGE-specific TCR T cells against tumor cells expressing the respective HLA restriction element with or without treatment with 0.5 μM DAC. Numbers under the bars indicate relative expression to housekeeping gene (%HKG) of indicated *MAGE* genes as determined by qPCR. Individual symbols indicate average values of technical duplicates of different donor T cells used to generate biological replicates of TCR-transduced T cells. Statistical comparisons show multiple t tests paired for T cell donors. (B) Reactivity of purified control CMV-specific TCR T cells against the tumor cell panel used in (A). Statistical comparisons show multiple t tests paired for T cell donors; *p* values are indicated underneath the bar plots. (C) Recognition of DAC- or DMSO-treated tumor cells expressing the respective HLA allele by allo-HLA reactive T cell clones. Statistical comparisons show results of paired t tests. Symbols represent average values of technical duplicates for cell lines indicated in the legend. (D) Expression of indicated cytokines or activation markers on MAGE-A1-specific TCR T cells after exposure to DMSO- or DAC-treated target cells. Statistics depict unpaired t test on technical replicates of one representative experiment of two. (E) IncuCyte-based killing assay of SK2.3 + HLA-B∗35:01 treated with DMSO (left) or with DAC (right). Normalized data to killing induced by allo-HLA-specific T cell clones. Each value represents the average of technical triplicates. Statistics depict one-way analysis of variance (ANOVA) comparing killing at the last time point (21 h) of technical triplicates. Data show one representative experiment of two.

Purified, MAGE-specific TCR T cell products were then assessed for antigen-specific cytokine secretion after overnight coculture with DAC- or DMSO-treated tumor cell lines expressing the respective restriction element (HLA) (Figure 2A). DAC treatment of tumor cells again resulted in the induction of *MAGEA1*, *MAGEA3/MAGEA6*, or *MAGEA9* expression as validated by qPCR. Generally, increase in *MAGE* expression correlated with recognition by MAGE-A1-, MAGE-A3/A6-, and MAGE-A9-specific TCR T cells. Notably, DAC treatment induced recognition of tumor cells that were not recognized by MAGE-specific TCR T cells when treated with DMSO only (e.g., MAGE-A1 vs. HCT116). When encountering tumor cells that were already recognized by MAGE-specific TCR T cells in the absence of DAC treatment, DAC treatment resulted either in a further increase in antigen-specific cytokine secretion (e.g., MAGE-A1 vs. MEL06.24) or did not further affect IFN-γ secretion (e.g., MAGE-A1 vs. MEL15.09b). Similar patterns were observed for all three specificities tested, although none of the tested tumor cells were recognized by MAGE-A9-specific TCR T cells unless treated with DAC. These effects were antigen-specific, as DAC treatment did not induce aberrant recognition of tumor cells by T cells expressing a TCR specific for a CMV-derived peptide in the context of HLA-A∗02:01 (Figure 2B). Furthermore, DAC treatment did not affect the recognition of most tumor cells by allo-HLA-reactive T cell clones (Figure 2C). In accordance with this observation, DAC treatment did not affect the expression of HLA-ABC and ICAM-1 (Figure S3).

To further profile the functional properties of MAGE-specific TCR T cells reacting toward DAC-treated cell lines, we analyzed the expression of cytokines and T cell activation markers. DAC-treated cell lines HCT116 and SK2.3 induced potent antigen-specific activation of MAGEA1 TCR T cells, as indicated by substantially increased production of IFN-γ, tumor necrosis factor alpha (TNF-α), and interleukin-2 (IL-2), as well as increased expression of CD137 and CD154 (Figures 2D and S4).

The DAC-mediated *de novo* reactivity as consequence of DAC-induced *MAGE* expression was confirmed in a cytotoxicity assay against the HLA-A∗01- and HLA-A∗02-positive melanoma cell line SK2.3 transduced to express HLA-B∗35 (Figure 2E). SK2.3 naturally expresses *MAGEA3/MAGEA6*, while *MAGEA1* and *MAGEA9* are not expressed. Without DAC treatment, SK2.3 + HLA-B∗35 was lysed by MAGE-A3/A6-specific TCR T cells, whereas MAGE-A1- and MAGE-A9-specific TCR T cells did not induce target cell lysis. After DAC treatment, SK2.3 + HLA-B∗35 was sensitized toward lysis by MAGE-A1- and MAGE-A9-specific TCR T cells, whereas it remained sensitive to lysis by MAGE-A3-/A6-specific TCR T cells. CMV-specific TCR T cells did not induce lysis of DAC-treated SK2.3 cells. Together these results indicate that DAC-induced *MAGE*-expression sensitized multiple cell lines toward antigen-specific recognition by MAGE-specific TCR-engineered T cells.

### *MAGE* upregulation after DAC treatment is not limited to tumor cells

**Figure 3:**
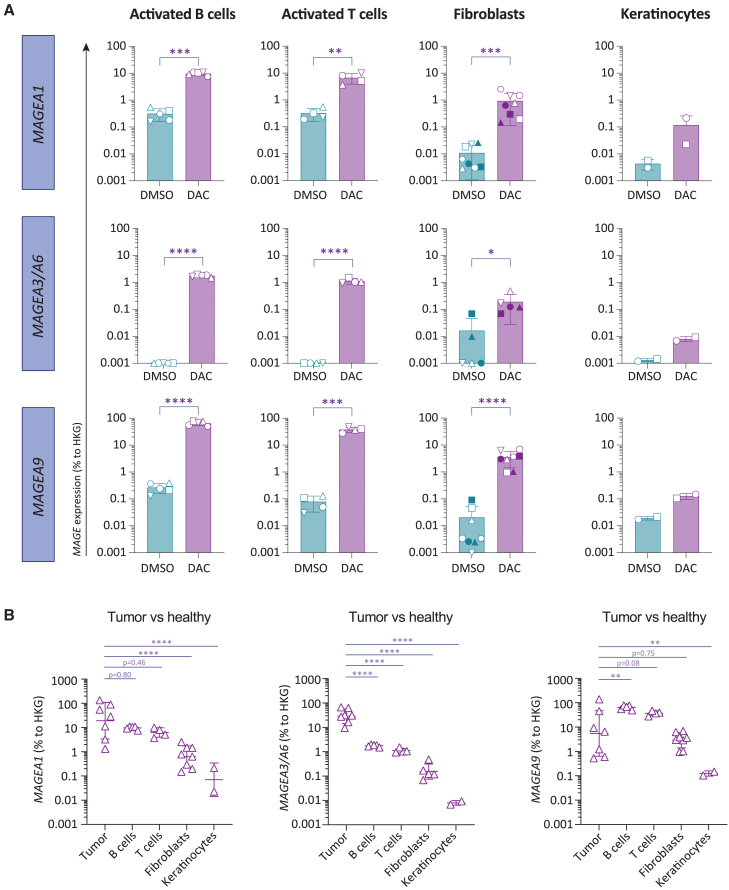
DAC treatment induces MAGE-A expression in healthy tissues (A) Expression of *MAGEA1*, *MAGEA3*/*MAGEA6*, or *MAGEA9* on human activated B cells or T cells, fibroblasts, and keratinocytes after DAC or DMSO treatment as determined by qPCR. Individual symbols depict biological replicates obtained from independent donors. Statistics depict paired t tests on log-transformed data. (B) Comparison of relative expression of *MAGEA1*, *MAGEA3*/*MAGEA6*, or *MAGEA9* between tumor cells and indicated healthy cells after DAC treatment. One-way analysis of variance (ANOVA) comparing tumor cells to healthy cell subsets on log-transformed data. Healthy cell subsets same data as in (A). See Table S1 for source data of tumor cells.

The assumption that DAC treatment selectively upregulates CTAs in tumor cells is based on the relative increased proliferative turnover of tumor cells. We therefore wondered whether DAC could also induce expression of *MAGE* genes in highly proliferating, healthy cells. To this end, we tested the effect of DAC treatment on *MAGE* expression in activated primary human T cells and B cells and in fibroblasts and keratinocytes. Both activated T cells and B cells have been described to undergo extremely rapid proliferation during peak expansion with doubling times of less than 12 h, whereas fibroblasts and keratinocytes typically display doubling times of more than 24 h during subconfluent *in vitro* cultures.^23^^,^^24^^,^^25^ Curiously, DAC treatment resulted in strong induction of *MAGEA1*, *MAGEA3/MAGEA6* as well as *MAGEA9* not only in activated B cells and activated T cells but also in fibroblasts (Figure 3A). *MAGE* expression in keratinocytes also showed a trend of increased expression.

To assess whether *MAGE* genes were induced to levels that could be relevant for T-cell-mediated recognition, we compared the relative expression levels of *MAGEA1*, *MAGEA3/MAGEA6*, and *MAGEA9* after DAC induction in healthy cells to the relative expression levels in tumor cells (Figure 3B). *MAGEA1* was induced up to 10% of housekeeping genes (HKGs) in healthy B cells and T cells, whereas in fibroblasts *MAGEA1* was induced up to around 1% of HKG expression. *MAGEA1* expression in healthy cells therefore falls into similar expression range as observed in tumor cells that displayed an expression range between 1% and 100% of HKG. For *MAGEA3/A6*, gene expression was induced to 1% of HKG for healthy B cells and T cells and around 0.1% for fibroblasts, which was generally lower than in tumor cells with a mean relative expression level of ∼30% HKG expression. *MAGEA9* showed the strongest induction in healthy B cells and T cells, reaching relative expression levels that were comparable or even higher than in the tumor cells tested. Keratinocytes generally displayed lower levels of expression of *MAGEA1*, *MAGEA3/MAGEA6* as well as *MAGEA9*. These results indicate that DAC-induced upregulation of *MAGE* expression is not tumor-specific. Instead, DAC can also induce substantial expression of *MAGEA1*, *MAGEA3/MAGEA6*, and *MAGEA9* not only in activated primary human T cells and B cells but also in fibroblasts.

### DAC treatment induces MAGE-specific reactivity against activated T and B cells

**Figure 4:**
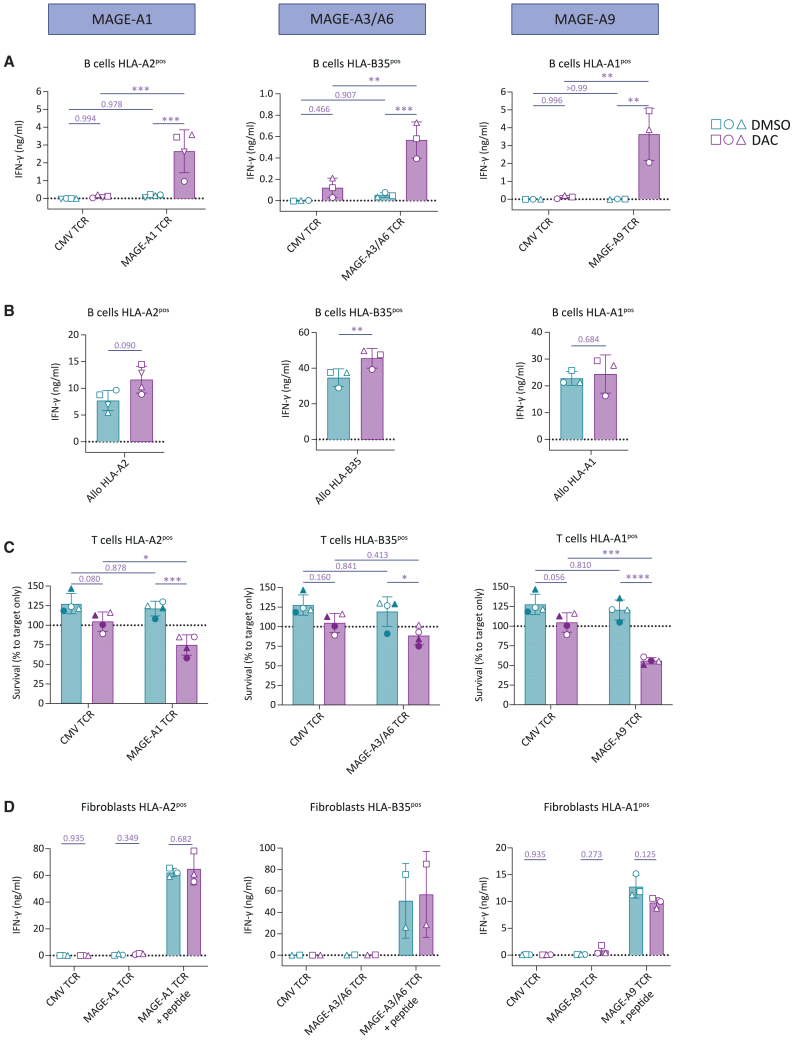
DAC treatment imposes potential on-target off-tumor toxicity by MAGE TCR T cells toward dividing healthy cells Activated B cells, activated T cells, or fibroblasts were treated with DAC or DMSO and assessed for their stimulatory capacity of indicated MAGE-A1-, MAGE-A3/A6-, or MAGE-A9-specific purified TCR T cells. (A) IFN-γ secretion of MAGE- or CMV-specific TCR T cells when encountering indicated B cells. Each symbol represents a biological replicate of cells isolated from a different healthy donor. Statistics depict results of a one-way analysis of variance (ANOVA). (B) IFN-γ secretion by indicated allo-HLA reactive T cell clones. Data obtained in the same experiment as shown in (A). Statistics depict results of a paired t test. (C) FACS-based quantification of DAC- or DMSO-treated T cell survival after overnight incubation with MAGE- or CMV-specific TCR T cells in an E:T ratio of 3:1. Each symbol represents a biological replicate of cells isolated from a different healthy donor. Statistics depict results of a one-way analysis of variance (ANOVA). (D) IFN-γ secretion of MAGE- or CMV-specific TCR T cells after overnight coculture with DAC- or DMSO-treated fibroblasts + or – MAGE peptide (250 nM). Each symbol represents a biological replicate of cells isolated from a different healthy donor. Statistics depict results of multiple paired t tests.

To investigate if the observed increased *MAGE* expression after DAC treatment of activated B cells and T cells and of fibroblast and keratinocytes would be sufficient to induce MAGE-specific T cell reactivity, we stimulated purified MAGE-A1-, MAGE-A3/A6-, or MAGE-A9-specific TCR T cells overnight with respective DAC-treated healthy target cells (Figure 4). Non-treated activated B cells were not recognized by any of the MAGE-specific TCR T cells (Figure 4A). After DAC treatment, however, MAGE-A1-, MAGE-A3/A6- as well as MAGE-A9-specific TCR T cells recognized B cells as indicated by IFN-γ secretion. The amount of IFN-γ correlated with the degree of induction of the respective MAGE protein targeted (MAGEA9 > MAGEA1 > MAGEA3/MAGEA6) (Figure 3B). DAC treatment did not result in T cell reactivity against target B cells lacking the respective restriction element (Figure S5). Furthermore, the recognition of B cells by allo-HLA-A2, -B35, and -A1 control T cell clones was already high, and either remained unaffected (allo-HLA-A1), or tended to increased (allo-HLA-A2, -B35) after DAC treatment (Figure 4B), resembling the reactivity of these allo-HLA control T cell clones toward tumor cells after DAC treatment (Figure 2C). Together, this indicates an HLA-restricted and antigen-specific effect of DAC treatment on the recognition of activated B cells by MAGE-specific TCR T cells.

To assess reactivity of MAGE-specific TCR T cells against other T cells, we assessed T cell fratricide of DAC-treated, non-edited activated T cells in a flow-cytometry-based killing assay. We observed induction of T cell fratricide of DAC-treated target T cells for both MAGE-A1- and MAGE-A9-specific TCR T cells, whereas recognition by MAGE-A3-/A6-specific TCR T cells showed a trend of increased antigen-specific loss of target T cell viability (Figure 4C). The degree of target T cell killing corresponded to the degree of DAC-mediated induction of the respective *MAGE* gene in T cells (*MAGEA9* > *MAGEA1* > *MAGEA3/MAGEA6*) (Figure 3B). Despite recognition of B cells and T cells, MAGE-specific TCR T cells did not recognize DAC-treated fibroblasts (Figure 4D), corresponding with a lower degree of *MAGE* induction after DAC treatment as compared to B cells or T cells (Figure 3B). In conclusion, DAC treatment was sufficient to induce MAGE expression in healthy B cells and T cells to a level that resulted in MAGE-specific TCR T cell recognition, warranting reevaluation of the statement that DAC exerts a tumor-restricted effect on the induction of CTAs.

## Discussion

In this study, we aimed to improve the recognition of tumor cells by CTA-specific T cells using demethylating agents to increase their antigen expression. DAC treatment mediated *MAGE* upregulation, resulting in improved recognition by MAGE-specific TCR T cells in multiple DAC-treated tumor cells derived from various different tumor types. Importantly, DAC treatment resulted in the recognition of tumor cells by MAGE TCR T cells that were previously not recognized. However, DAC also induced *MAGE* expression in activated T and B cells and to a lesser extent in fibroblasts. This resulted in MAGE TCR T cell recognition against DAC treated T and B cells corresponding with elevated *MAGE* expression levels.

In the setting of MAGE-specific TCR gene therapy, DAC treatment could potentially increase the number of patients who can be treated by inducing *MAGE* expression in patients with low or heterogeneous MAGE expression that would otherwise be ineligible for MAGE-specific TCR T cell therapy, making a combination of DAC treatment with MAGE-TCR gene therapy an attractive strategy.^10^ However, installment of *de novo* reactivity was not restricted to tumor cells but was also observed against healthy activated B cells and T cells, raising the question whether DAC treatment could result in unwanted on-target off-tumor toxicity when combined with MAGE TCR T cell therapy. The high *MAGE* upregulation in activated T and B cells can probably be explained by their high proliferative capacity, making activated T and B cells more sensitive for DAC incorporation into their DNA compared to cell subsets with lower proliferative capacity. In fibroblasts that generally display a lower proliferation rate, *MAGE* expression was also induced but not to an extent that resulted in reactivity of MAGE-specific TCR T cells. We hypothesize that reactivity against activated T and B cell subsets would not directly lead to life-threatening toxicity since rapidly proliferative T and B cells form only a small portion of the total T and B cell population. Depletion of actively proliferating B and T cells could however make patients more susceptible to infectious diseases, calling for careful monitoring of patients after T cell infusion.^26^^,^^27^ Additionally, the strong *MAGE* upregulation by DAC in quickly dividing cells might also indicate a potential risk for other normal tissues such as epithelial cells in the intestine. These concerns should be taken into consideration when combining DAC treatment with CTA-specific TCR gene therapy.

Previously, DAC therapy has been tested in cancer patients in combination with CTA vaccination. This led to clinical benefit of individual patients but induced only limited overall clinical effects.^28^^,^^29^ In those patients in whom clinical benefit was observed, no on-target off-tumor toxicity was observed, indicating that potential reactivity against healthy rapidly dividing cells did not cause a major problem in this setting. However, the absence of toxicity in this setting could also be explained by a lack of induction of high-avidity CTA-specific T cells through vaccination. This is different from TCR gene therapy, where CTA-specific T cells with a high functional avidity can be generated that might be potentially more sensitive toward antigen.

In the setting of MAGE-TCR T cell therapy, it might be advisable to temporally separate DAC treatment from therapeutic T cell infusion, i.e., administer MAGE-specific TCR cells only after DAC treatment. That way, it should be possible to minimize depletion of proliferating B and T cells and at the same to prevent fratricide of MAGE-specific TCR T cells. Furthermore, acute toxicity versus gut epithelial cells could also be minimized given their very short natural turnover time. In principle, avoiding simultaneous exposure to DAC- and MAGE-specific TCR T cells should not diminish the desired effect on tumor cells since DAC-induced demethylation in tumor cells was shown to be long-lasting. *De novo* expression of *MAGEA1* in melanoma cells was detectable up to 2–5 months after the end of DAC treatment.^30^^,^^31^

In summary, DAC treatment can substantially induce or enhance the reactivity of MAGEA1-, MAGEA3/A6-, and MAGEA9-specific TCR T cells toward diverse tumor cell lines. However, *MAGE* upregulation by DAC was not limited to tumor cell lines but was also observed in DAC-treated healthy cell subsets such as highly proliferating T and B cells. This could potentially result in on-target off-tumor toxicity, although unlikely to be life-threatening, and should be taken into consideration when treating patients with DAC prior to TCR gene therapeutic strategies. Overall, DAC treatment prior to MAGE-specific TCR gene therapy potentially enhances the anti-tumor efficacy and could make MAGE-specific TCR gene therapy available for a larger group of cancer patients, while potential *de novo* on-target off-tumor toxicities have to be taken into consideration.

## Materials and methods

### Cell culture and cell subset isolation

Tumor cell lines were cultured in Iscove’s modified Dulbecco’s medium (IMDM) (Lonza) supplemented with 10% fetal bovine serum (FBS) (Thermo Fisher Scientific), 1.5% L-glutamine (Lonza), and 1% penicillin/streptomycin (pen/strep) (Lonza). Early passage melanoma cell lines were cultured in Dulbecco’s modified eagle medium (DMEM) (Thermo Fisher Scientific) containing nonessential amino acids (NEAAs) with 7.5% FBS, 1.5% L-glutamine, and 1% pen/strep. Fibroblasts were cultured in DMEM supplemented with 10% FBS, 1.5% L-glutamine, and 1% pen/strep. Keratinocytes were cultured in keratinocyte serum-free medium (Gibco) supplemented with 0.2 ng/mL epidermal growth factor (EGF) (Promega), 30 μg/mL bovine pituitary extract (Thermo Fisher Scientific), 1.5% L-glutamine, and 1% pen/strep. Cell lines were transduced with HLA restriction elements where indicated.

Target T cells were isolated from peripheral blood mononuclear cells (PBMCs) by magnetic activated cell sorting (MACS) using anti-CD8 MicroBeads (Miltenyi Biotec). T cells (1 × 10^6^/mL) were stimulated with 1% TransAct (Miltenyi Biotec) and cultured in T cell medium (TCM) containing IMDM supplemented with 5% FBS, 5% human serum (Sanquin), 100 IU/mL IL-2 (Novartis Pharma), 1.5% L-glutamine, and 1% pen/strep.

B cells (CD19^+^ cells) were isolated from PBMCs by MACS using anti-CD19 MicroBeads (Miltenyi Biotec). B cells (0.3 × 10^6^/ml) were stimulated with 70 Gy irradiated CD40L^+^ mouse fibroblasts (1 × 10^6^/mL) and cultured in IMDM supplemented with 10% FBS, 1.5% L-glutamine, 1% pen/strep, and 2 ng/mL IL-4 (Schering-Plough). The LUMC ethical review board approved use of all the human material in this study (approval number B16.039). Materials were obtained after written informed consent in accordance with the Declaration of Helsinki.

### DAC treatment

DAC (Sigma-Aldrich) was dissolved in dimethyl sulfoxide (DMSO) (Emsure) and administered to the culture medium of each specific cell type. Negative control cells were treated with equal volumes of DMSO without DAC. Tumor cell lines and early passage melanoma cell lines were treated with 0.5 or 1 μM DAC, and adherent cells were at 50%–60% confluency at start of treatment.

Tumor cell lines, fibroblasts, and keratinocytes were DAC treated on day 0 and 3 and analyzed 6 days after start of treatment. For B cells and target T cells, DAC treatment (500 nM) was administered at day 3 and day 6 after initial stimulation. B cells and target T cells were analyzed for gene expression or included in co-culture assays on day 9 after stimulation.

### Quantitative real-time PCR

Total RNA was isolated from 0.1–5 × 10^6^ target cells using the Reliaprep RNA cell mini prep system according to manufacturer’s protocol (Promega). Moloney murine leukemia virus reverse transcriptase and Oligo (dT) primer (Invitrogen by Thermo Fisher Scientific) was used to convert total RNA to cDNA. qPCR was performed using Fast Start TaqDNA Polymerase (Roche) and EvaGreen (Biotum). Gene expression was measured on the Lightcycler 480 (Roche) using respective forward and reverse primers (Table S2). Target gene expression was calculated relative to the expression of HKG *GUSB*, unless specified differently in the figure legend.

### MAGE-A1 flow cytometry

To analyze protein expression of MAGE-A1 cell lines, HCT116 and SK2.3 were treated with 0.5 μM DAC or DMSO according to the established treatment regimen. Cells were fixed and permeabilized using the Foxp3/Transcription Factor Staining Buffer Set (Invitrogen). Cells were stained in 50 μL permeabilization buffer containing an unlabeled anti-MAGE-A1 (ABCAM, clone MA454) antibody. Cells were washed with PBS containing 0.8 mg/mL albumin (FACS buffer) and stained with the secondary antibody GAM-AF647 (Invitrogen). Cells were washed and measured on the BD LSRFortessa Cell Analyzer (BD biosciences).

### TCR gene transfer to human T cells

In this study, the following MAGE-specific TCRs were used: the MAGE-A1 TCR 4F7 specific for the KVLEYVIKV peptide presented in HLA-A∗02:01, the MAGE-A3/-A6 TCR 2H9 specific for the EVDPIGHLY/EVDPIGHVY peptides presented in HLA-B∗35:01, the MAGE-A9 TCR 2D8 specific for the YVGKEHMFY peptide presented in HLA-A∗01:01, and the CMV TCR specific for pp65 NLVPMVATV peptide presented in HLA-A∗02:01.^22^^,^^32^ The variable TCR-α and TCR-β fragments of the different TCRs were codon optimized and combined with codon-optimized and cysteine-modified murine TCR-αβ constant domains. The TCR chains were linked by a P2A sequence and cloned into an MP71 retroviral expression vector. To generate retroviral supernatants, Phoenix-A (ATCC) were transfected with plasmid DNA complexed with Fugene HD transfection reagent (Promega) and opti-MEM medium (Invitrogen Gibco). Retroviral supernatant was harvested 48 and 72 h after transfection before cryopreservation at −80°C.

Primary CD8^+^ T cells for TCR gene transfer were isolated from PBMCs by MACS using anti-CD8 Microbeads (Miltenyi Biotec). Isolated T cells (0.3 × 10^6^ cells/mL) were stimulated with irradiated (50 Gy) autologous PBMCs (1 × 10^6^ cells/well) and 0.8 μg/mL PHA and cultured in TCM. On day 2, thawed retroviral supernatants were added to 24-well non-tissue cultured treated plates (Greiner Bio-One) precoated with RetroNectin (Takara) and blocked with 2% human serum albumin (Sanquin). Retroviral supernatant was spun down for 20 min, 2,000 g at 4°C, after which the virus supernatant was removed, and 0.3 × 10^6^ CD8^+^ T cells were added for overnight culture. T cells were harvested, transferred to 24-well culture plates (Costar), and further expanded. Five days after transduction, transduced T cells were enriched by MACS using APC-conjugated anti-mouse TCR-β chain (mTCR) antibody (BD biosciences) and anti-APC Microbeads (Miltenyi Biotec). pHLA tetramer and mTCR staining followed by flow cytometry were assessed to confirm cell surface expression of the TCRs, and TCR T cells were used for functional assay 8–10 days after initial isolation.

### T cell reactivity assays

The reactivity of TCR T cells toward DAC-treated cells was investigated by measurement of IFN-γ production after overnight co-culture. Five thousand T cells were co-cultured with respective tumor cell lines and healthy cell subsets in an effector:target (E:T) ratio of 1:2 (fibroblasts and keratinocytes) or 1:4 (tumor cell lines and B cells) in 60 μL TCM per well in 384-well flat-bottom culture plates (Greiner Bio-one). To induce HLA expression on adherent cell lines, 100 IU/mL IFN-γ (Boehringer Ingelheim) was added to the cultures 48 h prior to coculture with T cells. Where indicated, target cells were peptide pulsed for at least 30 min at 37°C with 250 nM respective peptide. After overnight co-culture recognition of target cells was determined by measuring IFN-γ production in the supernatant by ELISA (Sanquin/Invitrogen/Diaclone). The supernatant was diluted 5, 25, and 125 times to determine concentrations based on the linear part of the standard curve. The Hamilton Microlab STAR Liquid Handling System (Hamilton company) was used to transfer supernatants from the culture plates to the high-binding 384-well ELISA plates (Greiner Bio-one). ELISA plates were washed extensively between the coating, blocking, detection, and readout steps with a Zoom HT LB 920 Microplate Washer (Berthold).

### T cell activation analysis

To analyze T cell activation and cytokine production, 50,000 DAC- or DMSO-treated tumor cells were seeded on a flat bottom 96-well plate. The following day MAGE-A1 TCR (4F7) T cells and control CMV TCR T cells enriched for mTCR were co-cultured in a 1:1 ratio 10 days after activation. After O/N co-culture the stimulation was stopped by washing the cells in PBS followed by viability staining using Zombie-Red (BioLegend). After washing with PBS, cells were fixed and permeabilized using the FOXP3 buffer set (Invitrogen). Cells were stained using 20 μL antibody staining mix containing Brilliant Stain Buffer Plus (BD Biosciences), 0.8 mg/mL albumin, and antibodies directed against CD3, CD4, CD8, TNF-α, IFN-γ, IL-2, CD154, and CD137 followed by incubation for 30 min at room temperature. Cells were washed and resuspended in FACS buffer and measured on a 5-laser aurora (Cytek Biosciences). Fluorochrome, clone, supplier, and catalog number information of antibodies used in these experiments can be found in Table S3.

### IncuCyte cytotoxicity assay

DMSO- or DAC-treated SK2.3 cells (10,000) transduced to express HLA-B∗35 were plated in 384-well plates and allowed to adhere overnight at 37°C. The following day, 5,000 transduced purified transduced CD8^+^ T cells were added. Cells were imaged in an IncuCyte S3, and integrated intensity of propidium iodide (PI) (RCU × μm^2^/Image) was acquired every hour for a total of 21 h. Killing was calculated based on background-corrected measured PI RCU normalized to PI RCU achieved with an allo-HLA reactive T cell clone (positive control). Statistics were calculated on experimental triplicates using one-way analysis of variance (ANOVA) comparing killing at the last time point (21 h).

### FACS-based cytotoxicity assay

T cell fratricide was assessed using a flow-cytometry-based cytotoxicity assay. To generate target T cells, PBMCs were stimulated using transact and IL-2. On day 3 and day 6 after stimulation, T cells were treated with 0.5 μM DAC or DMSO. On day 9 after stimulation (day 6 after first DAC treatment), 20,000 target T cells were incubated with 60,000 transduced and purified MAGE-specific TCR-engineered T cells. After overnight coculture, target T cell survival was quantified using isovolumetric flow cytometry. Target T cells were discriminated from allogeneic TCR-engineered T cells based on HLA-A∗02 and/or HLA-B∗07 expression. The following flow cytometry panel was used: live/dead sytox blue, HLA-B∗07 APC, HLA-A∗02 FITC, CD19 PE-vio615, CD8 AF700, CD4 PE-Cy7.

### Statistical analysis

Statistical analysis was performed using GraphPad Prism9 software. Respective statistical tests used are indicated in figure legends. The definition of statistical significance was set at *p* < 0.05. Significance levels are defined as ∗*p* < 0.05, ∗∗*p* < 0.01, ∗∗∗*p* < 0.001, and ∗∗∗∗*p* < 0.0001. Throughout the manuscript error bars represent standard deviations.

## Data availability

The data that support the findings of this study are available from the corresponding author, M.H.M.H., upon reasonable request.

## Acknowledgments

The authors thank the operators of the Flow cytometry Core Facility (FCF) of Leiden University Medical Center (LUMC) in Leiden, the Netherlands for cell sorting assistance. This study was funded by Bellicum Pharmaceuticals (unrestricted grant) and Health-Holland (grant number LSHM17002).

## Author contributions

M.A.J.d.R., conceptualization, formal analysis, investigation, methodology, verification, visualization, writing—original draft, and writing—review & editing. M.H.M., formal analysis, investigation, methodology, verification, and visualization. A.K.W., formal analysis, investigation, and methodology. D.F.G.R., formal analysis, investigation, and methodology. R.S.H., investigation. D.M.v.d.S., investigation. E.M.E.V., resources. T.L.A.W., formal analysis, visualization, writing—original draft, and writing—review & editing. J.H.F.F., conceptualization, supervision, and writing—review & editing. M.H.M.H., conceptualization, project administration, resources, supervision, and writing—review & editing.

## Declaration of interests

M.H.M.H. and J.H.F.F. are co-inventors on a patent describing MAGE T cell receptors (T cell receptors directed against melanoma-associated antigen and uses thereof). PCT/NL2023/050325, filed June 10, 2022.

## Supplemental information

Document S1. Figures S1–S5 and Tables S1–S3

Document S2. Article plus supplemental information
