# Supplementary material for: DNA-demethylation by DAC induces MAGE expression and MAGE-specific T cell reactivity against tumors but also healthy cell subsets
Source: Mol Ther Oncol. 2025 Jul 17;33(3):201018. doi: 10.1016/j.omton.2025.201018 (PMC12335964; doi:10.1016/j.omton.2025.201018)
Supplement: Document S1. Figures S1–S5 and Tables S1–S3 [file mmc1.pdf]

OMTON, Volume *33*

Supplemental information

DNA-demethylation by DAC induces *MAGE* expression and MAGE-specific T cell reactivity against tumors but also healthy cell subsets

Marije A.J. de Rooij, Miranda H. Meeuwsen, Anne K. Wouters, Dennis F.G. Remst, Renate S. Hagedoorn, Dirk M. van der Steen, Els M.E. Verdegaal, Tassilo L.A. Wachsmann, J.H. Frederik Falkenburg, and Mirjam H.M. Heemskerk

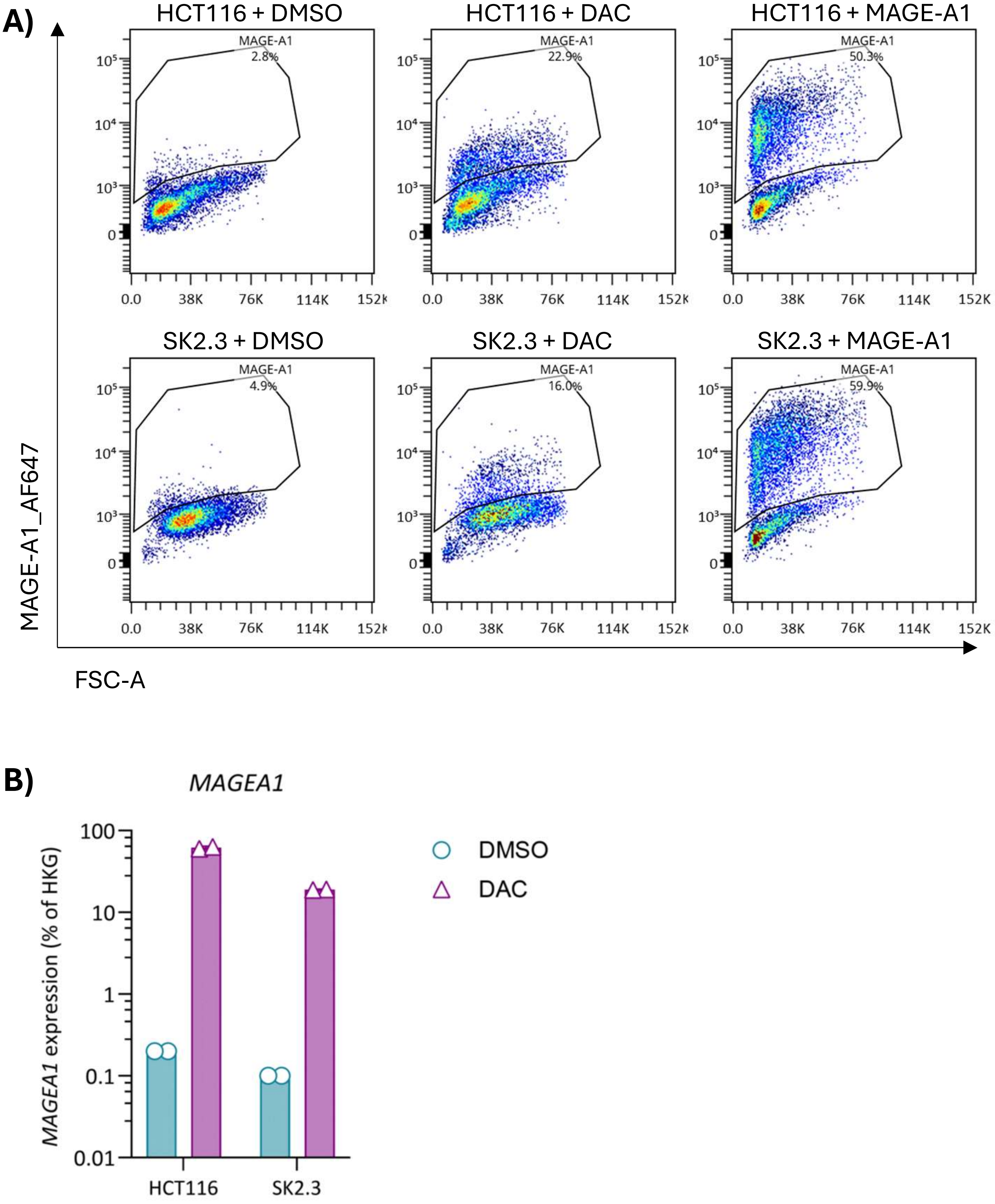

Figure S1. Analysis of MAGE-A1 protein after DAC treatment. A) Raw flow cytometry plots of intracellular MAGE-A1 staining in HCT116 and SK2.3 cells treated with DMSO (left) or 0,5uM DAC (middle). As a positive control HCT116 and SK2.3 transduced with MAGE-A1 mixed in a 1:1 ratio with untransduced cells were included. Cells were gated on live cells. Data originates from one of the experiments shown in Figure 1E. B) *MAGEA1* gene expression relative to house keeping gene was analyzed using qPCR. Cells obtained from two of the three experiments shown in Figure 1E were analyzed.

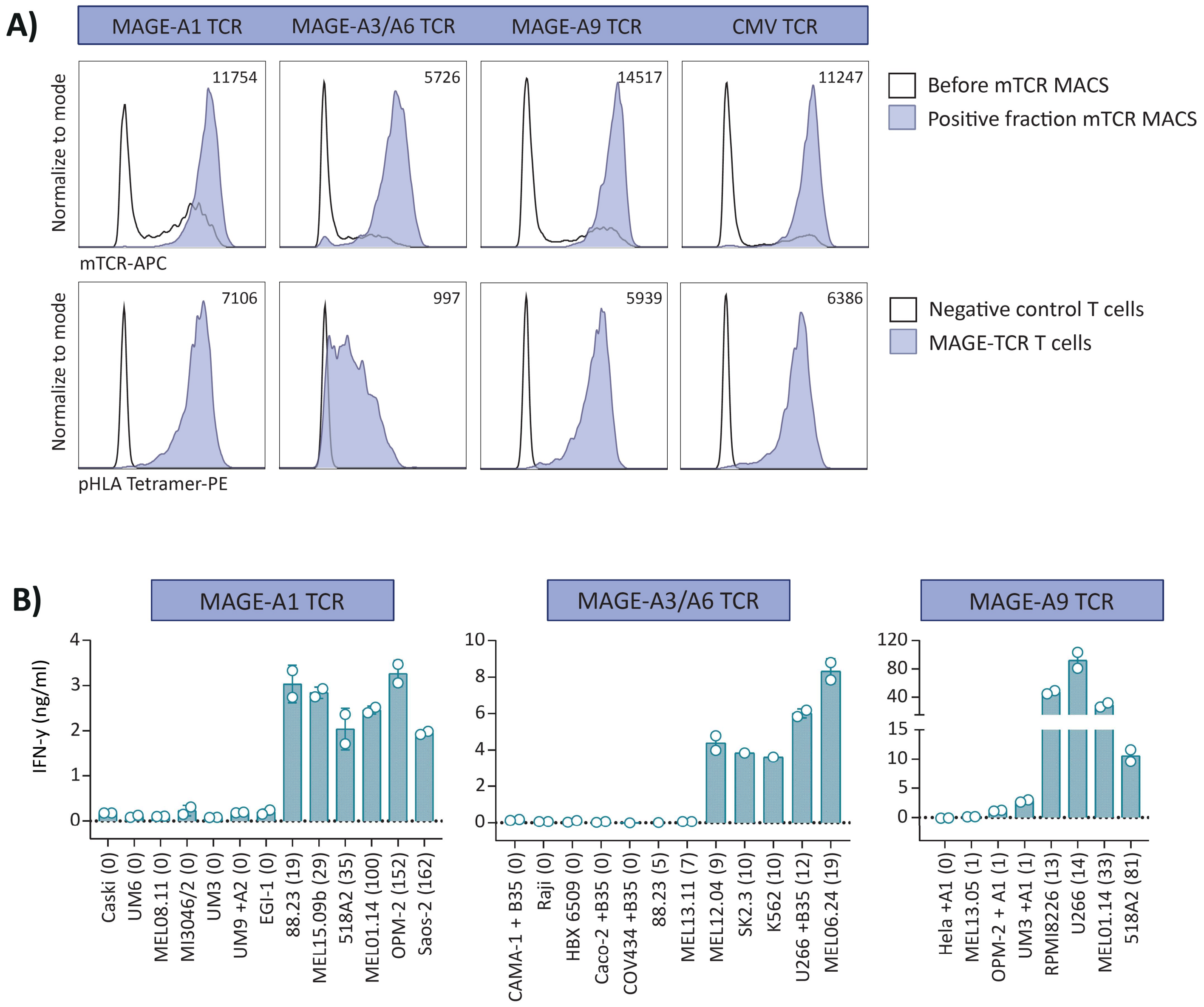

Figure S2. Generation and validation of MAGE-specific TCR T cell products. Primary human CD8 T cells were activated and retrovirally transduced to express constant domain murinized MAGE-specific or control CMV-specific TCRs. A) (above) FACS plots showing expression of indicated transduced TCRs before (black/white) and after (blue) MACS enrichment for mTCR. (below) Tetramer staining of indicated purified TCR transduced T cells. B) Reactivity of purified MAGE-specific TCR T cells against a panel of antigen positive and antigen negative target cells. Expression of respective *MAGE* genes is indicated as % of housekeeping genes *GUSB*, *PSMB4* and *VPS29* next to the names of the cell lines. Data points represent technical duplicates.

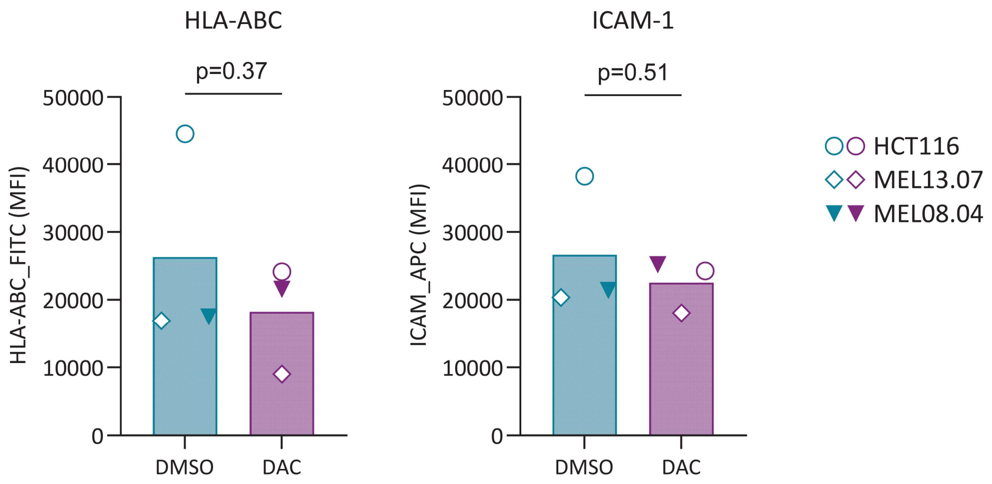

Figure S3. Analysis of HLA and ICAM-1 protein after DAC treatment. Tumor cells lines treated with DAC or DMSO as a control were stained for HLA-ABC (left) and ICAM-1 (right) and analyzed by flow cytometry. Groups were statistically compared using paired T-tests. Experiment was performed once.

A)

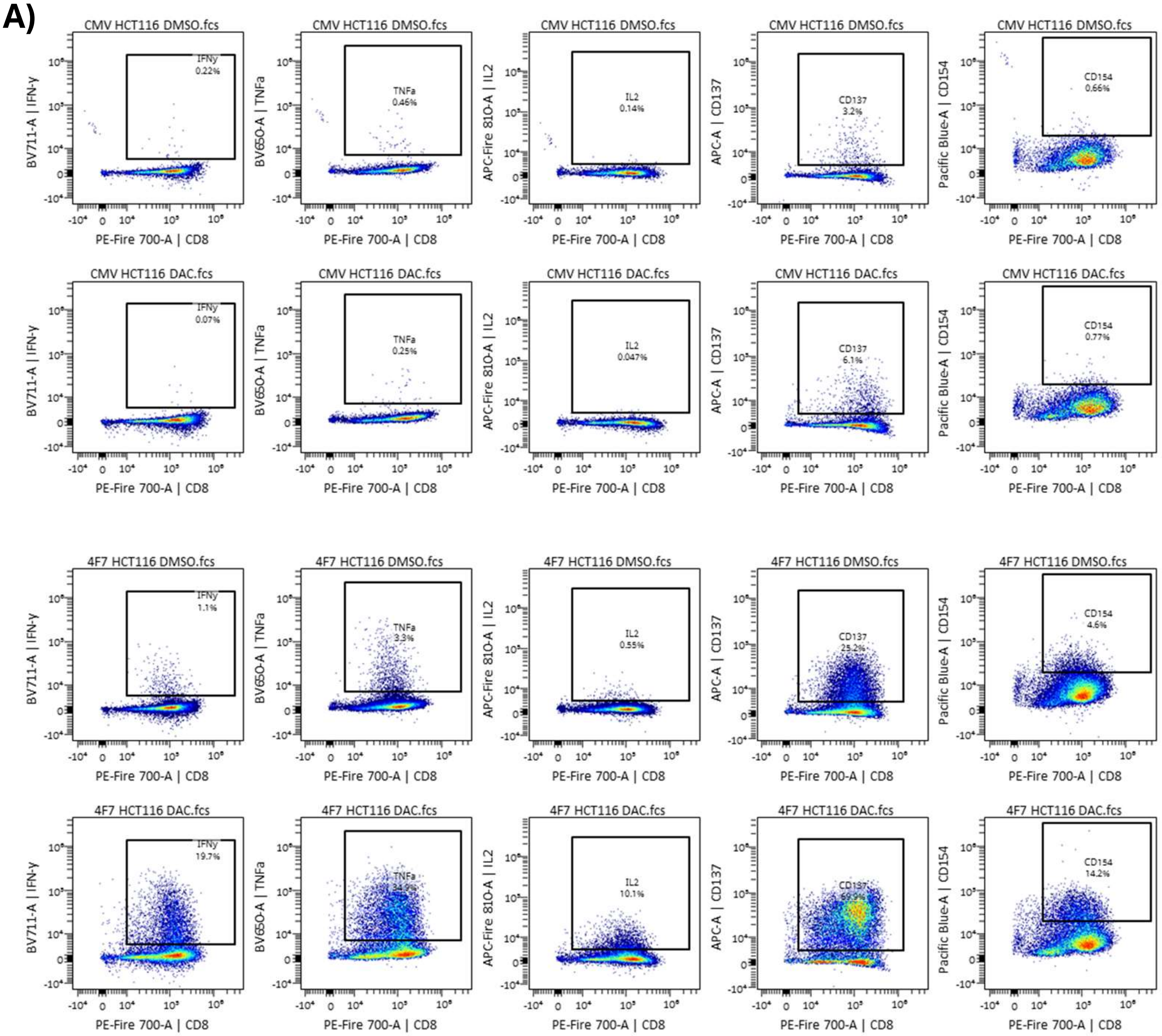

B)

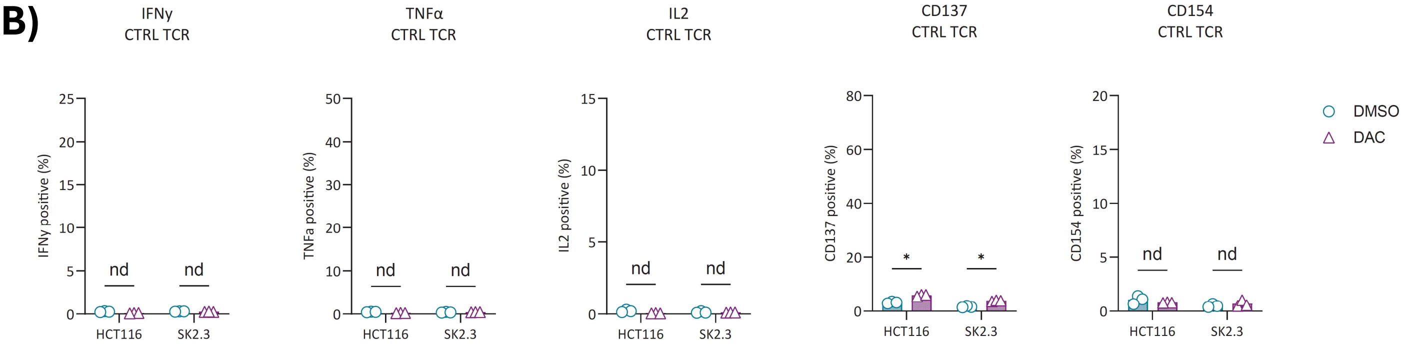

Figure S4. Analysis of activation state of MAGE-A1 TCR T cells after co-culture with DAC treated tumor cell lines. A) Raw flow cytometry plots of Control CMV-TCR T cells (top two rows) or MAGE-A1 targeting TCR-4F7 T cells (bottom two rows) after overnight co-culture with HCT116 cells pre-treated with DMSO as a negative control (first and third row) or DAC (second and fourth row). Cells were fixed, permeabilized and stained for cytokines IFNγ, TNFα, IL-2 and activation marker CD137 and CD154.
This data originates from the experiment shown in Figure 2D. B) Summary of cytokine and activation marker staining on control CMV TCR (CTRL TCR) T cells after co-culture with DMSO and DAC treated HCT116 or SK2.3 cells. Data obtained in the same experiment as in Figure 2D/Supplemental figure 4A. Symbols represent technical triplicates. Groups were compared using unpaired T-tests. Experiment representative of two independent experiments.

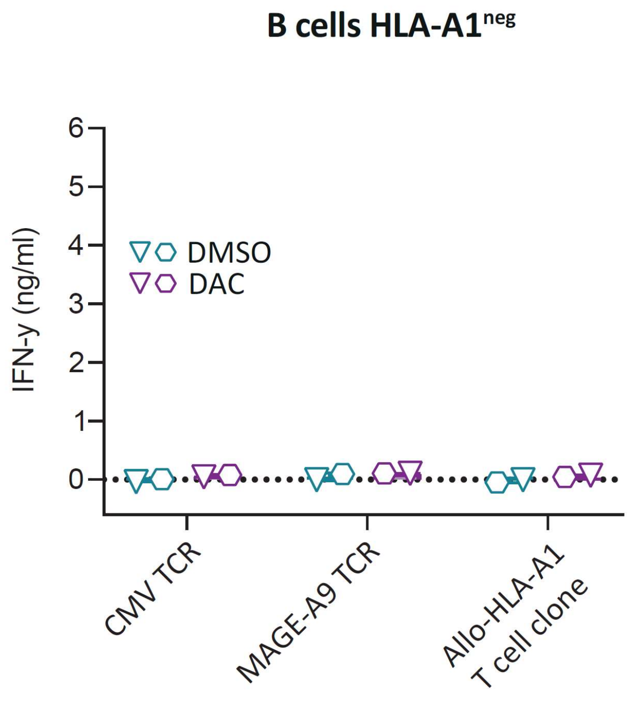

Figure S5. HLA-A1 restricted MAGE-A9 specific TCR T cells do not react against DAC treated activated B cells that are negative for HLA-A1. Activated B cells that do not express HLA-A1 were treated with DMSO or DAC and incubated overnight with of HLA-A1 restricted MAGE-A9 specific TCR T cells or indicated control T cells. IFN-γ secretion was assessed by ELISA.

Table S1. Source data of figure 3B depicting expression of indicated *MAGE* genes normalized to expression of housekeeping genes (in %).

	MAGEA1	*MAGEA3/A6*	*MAGEA9*
HCT116 WT + DAC	135,19	66,90	138,99
WiDr WT + DAC	55,48	30,89	45,53
MI 3046/2 WT + DAC	28,52	9,54	9,28
SK2.3 + HLA-B35 + DAC	11,23	31,75	6,52
MEL13.07 WT + DAC	3,26	27,64	0,53
MEL15.09b WT + DAC	90,13	16,44	0,60
MEL06.24 WT + DAC	1,32	62,63	1,24

Table S2: Primers used for RT-qPCR experiments

Gene	Forward primer	Reverse primer
MAGE-A1	GAGTCCTTGTTCCGAGCAGT	GGCTCCCTGGCTCGATATTT
MAGE-A3/A6	CCTGAGCAACGAGCGACG	TCAGAACCTTGCCTCCTCACC
MAGE-A9	GATCCTGCGCACTACGAGTT	ATGGGTAGCAGATGGGCTCT
GUSB	ACTGAACAGTCACCGACGAG	GGAACGCTGCACTTTTTGGT
PSMB4	GTTTCCGCAACATCTCTCGC	CATCAATCACCATCTGGCCG
VPS29	TGAGAGGAGACTTCGATGAGAATC	TCTGCAACAGGGCTAAGCTG

Table S3. Fluorochrome, supplier, clone and catalogue number (CAT#) information of antibodies used in T cell activation experiments

Marker	Fluorochrome	Supplier	Clone	CAT #
CD4	PE-fire 810	Biolegend	SK3	344677
CD8	PE-fire 700	Biolegend	SK1	344766
CD3	RB545	BD Biosciences	UCHT1	569197
IFN-γ	BV711	BD Biosciences	B27	564039
IL-2	APC-Fire 810	Biolegend	MQ1-17H12	500356
TNF-α	BV650	BD Biosciences	MAb11	563418
CD137	APC	BD Biosciences	4-1BB	550890
CD154	Pacific Blue	Biolegend	24-31	310820
zombie red	zombie red	Biolegend		423109
